# The cumulative social adversity hypothesis of psychosis: Intolerance of uncertainty and aberrant salience mediate the association between humiliation and psychotic-like experiences

**DOI:** 10.3389/fpsyt.2025.1647155

**Published:** 2025-11-07

**Authors:** Tomasz Bielawski, Błażej Misiak

**Affiliations:** Department of Psychiatry, Wroclaw Medical University, Wroclaw, Poland

**Keywords:** psychotic like experiences, intolerance of uncertainty, social defeat, humiliation, aberrant salience

## Abstract

**Background:**

The social defeat hypothesis, here framed as cumulative social adversity (CSA) to avoid disempowering terminology, posits that individuals with long-term experience of an unwanted, subordinate position present an elevated risk of psychosis. It has been observed that humiliation might be the most central component of the CSA hypothesis that increases the risk of psychosis through specific information processing patterns. The present study aimed to further investigate as to whether two cognitive processing patterns, i.e., aberrant salience (AS) and intolerance of uncertainty (IU), play a mediating role in the association between CSA and psychotic-like experiences (PLEs).

**Methods:**

A total of 1308 non-clinical young adults (aged 31.1 ± 5.9 years, 47.9% men) were assessed with self-reports recording the occurrence of PLEs, AS, IU, and cumulative humiliation via computer-assisted web interview over a 6-month period. A theory-driven, serial mediation model was analyzed.

**Results:**

Humiliation was not directly associated with the level of follow-up PLEs. However, two mediation paths linking humiliation and PLEs were statistically significant after adjustment for age, gender, education, monthly income, and baseline depressive symptoms. The first one led through AS (without a mediating effect of IU) and the second one led through IU and AS (a serial mediation). The indirect association of humiliation with PLEs through a mediating effect of IU (i.e., without AS) was not statistically significant.

**Conclusions:**

Our findings suggest that cognitive processing patterns, such as AS, and to a lesser extent IU, may serve as important psychological mechanisms through which cumulative humiliation may lead to the occurrence of PLEs.

## 1 Introduction

Psychosis exists on a continuum, with a range of experiences that can be observed even in healthy individuals (1). These subclinical phenomena, observed in healthy individuals, are commonly referred to as psychotic-like experiences (PLEs). They cover perceptual abnormalities and delusion-like experiences that show low severity and impact on general functioning. Their lifetime prevalence has been estimated at 6% and in most individuals they occur as transient experiences (2). Nonetheless, there is evidence that PLEs might precede the onset of psychosis, but might also occur in the context of various mental disorders that do not represent the psychosis spectrum (3, 4). The etiology of PLEs and possible contribution to the development of mental disorders are still unclear.

Several factors have been implicated in the development of PLEs, i.e., psychosocial factors, a history of childhood trauma, and cognitive processing differences (3). Psychosocial factors that might influence psychosis development via dopaminergic disruptions are conceptualized within the social defeat hypothesis (5). Considerding the fact that the term “social defeat” might be disempowering towards excluded groups, in this article we propose the term cumulative social adversity hypothesis of schizophrenia (CSA). We aim to retain continuity with the original model developed by Selten (5) highlighting the role of persistent exclusion, marginalisation, and subordination in sensitising dopaminergic pathways, while avoiding language that implies individual weakness. In accordance with the CSA framework, individuals who feel vulnerable in social interactions (6), may be more prone to develop cognitive processing differences (7) and increased levels of anxiety (8) that ultimately lead to the development of PLEs (9). Individuals whose sense of self naturally varies across contexts and who engage with others’ mental states in ways that differ from typical patterns may be more inclined to interpret certain interpersonal situations as humiliating. Such interpretations could, in turn, activate increased threat-detection processes and contribute to the occurrence of PLEs (6). Perhaps more importantly, humiliation can be enhanced by prolonged subordinate social position due to migration, low income, or minority status (10). In our most recent study, we found that cumulative humiliation may be the most central aspect of CSA that is most closely related to the occurrence of PLEs (11).

Intolerance of uncertainty (IU) refers to a negative cognitive response to ambiguity, characterized by the tendency to perceive possibility of a negative event as unacceptable and threatening, regardless of its actual likelihood (12). The construct is grounded in the premise that IU may contribute to the development of anxiety, which can be conceptualized as a complex preparatory response to potential, yet unidentified, threats (13–16). Importantly, uncertainty itself may be experienced as threatening, thereby intensifying anxiety and fostering a false sense of certainty regarding threat presence (12, 17). Although IU is recognized as a transdiagnostic risk factor for a range of mental disorders (18, 19), it has been most extensively examined in the context of anxiety disorders and depression (12, 14, 19–22, 60). Moreover, evidence suggests that IU is linked to heightened threat generalization and may facilitate early, automatic detection of ambiguous stimuli, accompanied by alterations in cognitive processing (14, 23). Individuals with high IU may be more likely to draw premature conclusions and adopt rigid beliefs when confronted with ambiguous information, potentially increasing the risk of PLEs (24, 25) and exaggerated assignment of salience (26). This is consistent with the established link between enhanced anxiety and PLEs development that often co-occur during the prodromal phase of psychosis (27, 28). In line with this, recent literature identifies IU as a potential transdiagnostic marker for assessing paranoia across clinical and subclinical populations (19). IU and paranoia may interact to exacerbate levels of negative affectivity and depressive symptoms. IU might therefore be one of the key transdiagnostic dimensions that binds and modulates different symptoms across anxiety, depression, and schizophrenia-spectrum conditions (15, 19, 29). Importantly, this raises the question of whether IU modulates the cognitive, somatic, and behavioral processes underlying anxiety, depression, and psychosis. Supporting this possibility, negative affect and worry (core processes closely linked to IU) have been implicated in both the emergence and exacerbation of psychotic symptoms (30, 31). Consistent with this view, a recent meta-analysis reported elevated IU in individuals meeting criteria for an ‘at-risk mental state’ for psychosis, as well as correlations between IU and psychotic symptoms involving delusions and paranoia in both clinical and nonclinical samples (29).

Aberrant salience (AS), defined as the inappropriate assignment of significance to otherwise innocuous stimuli (32, 33), may provide an important link between IU and the development of PLEs. Transient episodes of AS can occur in healthy individuals (34), but more pronounced and persistent AS increases the likelihood of overt psychotic symptoms over time (7). Alterations in perceived meaning and significance are core features in the onset of psychosis (35–37), and both AS and psychotic symptoms have been linked to similar disruptions in dopamine synthesis (38). AS also plays a central role in theoretical models of psychosis development (39). In our previous work, we found that AS fully mediated the relationship between cumulative humiliation and PLEs in healthy individuals (11).

Given its role in psychosis models, AS may represent the pathway through which elevated IU contributes to PLE development. This proposed sequence—where IU heightens vulnerability to AS-related processing differences—warrants direct empirical testing. The present study examines this possibility by modeling the relationships among humiliation, IU, AS, and PLEs in a single framework. Using serial mediation analysis in a non-clinical sample of young adults, we hypothesize that IU and AS function as mediators linking humiliation to PLEs.

## 2 Material and methods

### 2.1 The sample

The study was conducted by means of online surveys. Invitations were sent through the online platform designed for research surveys, maintained by the polling company (Pollster). Recruitment procedures were implemented in June 2024 and the surveys were conducted using computer-assisted web interview CAWI. The inclusion criteria were age between 18 and 40 years and a negative lifetime history of psychiatric treatment. An age restriction has been implemented, as psychotic disorders most often emerge in younger populations (40, 41). Additionally, participants were selected to reflect the sociodemographic characteristics of the Polish population based on data from 2021. Participants were reassessed after 6 months (June 2024). Some findings from the present cohort were published previously (11). The study received approval of the Bioethics Committee at Wroclaw Medical University, Wroclaw Poland (approval number: 22/2024).

### 2.2 Assessments

The participants were assessed with respect to the level of humiliation, AS and PLEs (at baseline and after 6 months) as well as IU (after 6 months). To maintain data reliability, several accuracy measures were integrated into the survey process, both during and after its completion. Participants who did not meet the predetermined accuracy standards were excluded from the final dataset. The exclusion criteria were: excessively short survey completion times (below 30% of the median completion time), failure to pass attention checks (i.e., participants were asked to respond to items requesting them to select a specific answer), inconsistent responses to repeated items, and the presence of random or nonsensical character strings in their responses.

#### 2.2.1 Humiliation

We used the Humiliation Inventory to assess the internal experience of humiliation (42). The inventory consists of 32 self-reported items rated between 1 (“not harmed at all”) to 5 (“extremely harmed”). The original version of this questionnaire includes two subscales, i.e., the cumulative humiliation subscale and the fear of humiliation subscale. The first one measures the severity of lifetime humiliating experiences, while the latter one records the level of anticipation and anxiety regarding future humiliating experiences. In our study, we used the first subscale that is based on 12 items (lifetime experiences of humiliation, e.g., “*Throughout your life how seriously have you felt harmed by being excluded?… by beeing cruelly criticized?… discounted?*”). The total score ranges between 12 and 60, where higher scores reflect higher levels of humiliation experiences. The Cronbach’s alpha was 0.961 in the present study.

#### 2.2.2 AS

The Aberrant Salience Inventory (ASI) was used to measure the tendency to assign meaning to irrelevant stimuli. The ASI consists of 29 self-report items(e.g., “*Do you sometimes notice small details that you have not noticed before that seem important?*”, “*Do you ever feel the need to make sense of seemingly random situations or occurrences?*”). with yes-or-no responses (rated as 1 or 0). It is based on five subscales (increased significance, senses sharpening, impending understanding, heightened emotionality, and heightened cognition) and has good psychometric properties (32). It has been shown that a higher ASI total score is related to a greater risk of psychosis (7, 35).The Cronbach’s alpha for ASI was 0.925 in the present study.

#### 2.2.3 PLEs

We used the Prodromal Questionnaire-16 (PQ-16) to record the presence of PLEs over the preceding month (43). The PQ-16 has been designed to detect psychosis risk states. It consists of 16 true-or-false items that capture various PLEs(e.g., “*I often seem to live through events exactly as they happened before (déjà vu)*”, “*I often hear unusual sounds like banging, clicking, hissing, clapping or ringing in my ears*”), along with associated distress rated from 0 (lack of distress) do 4 (significant distress). Two items (i.e., items 1 and 7) might measure depressive and anxiety symptoms. Therefore, we limited the analysis to 14 remaining items with the total score ranging between 0 and 14. In the present study, the Cronbach’s alpha for the presence subscale was found to be 0.844.

#### 2.2.4 Depressive symptoms

Due to the fact that PLEs are widely perceived as transdiagnostic phenomena, the present study also recorded the occurrence of depressive symptoms. To assess the levels of depressive symptoms(e.g., “*Over the last 2 weeks, how often have you been bothered by feeling tired or having little energy?… by little interest or pleasure in doing things?*”), the Patient Health Questionnaire-9 (PHQ-9) was administered (44). It records the level of depressive symptoms experienced over the preceding two weeks using a four-point scale. Responses to each item range from 0 – “not at all” to 3 – “nearly every day”. The overall score ranges between 0 and 27 (higher scores correspond with greater levels of depressive symptoms). In this study, Cronbach’s alpha of the PHQ-9 was 0.875.

#### 2.2.5 Intolerance of uncertainty

We used a short version of the Intolerance of Uncertainty Scale (IUS), consisting of 12 items (12). Each item(e.g., “*I can’t stand being taken by surprise*”, “*Uncertainty keeps me from living a full life*”) is rated on a Likert scale ranging from 1 (“not at all characteristic of me”) to 5 (“entirely characteristic of me”). The overall score ranges between 12 and 60. The original version of IUS was based on 27 items and was developed by Freestone and colleagues (1997). A 12-item version has been found to show a stable two-factor structure, representing both anxious and avoidance components of IU (45). In this study, the Cronbach’s alpha of the IUS was 0.901.

### 2.6 Data analysis

**Figure 1:**
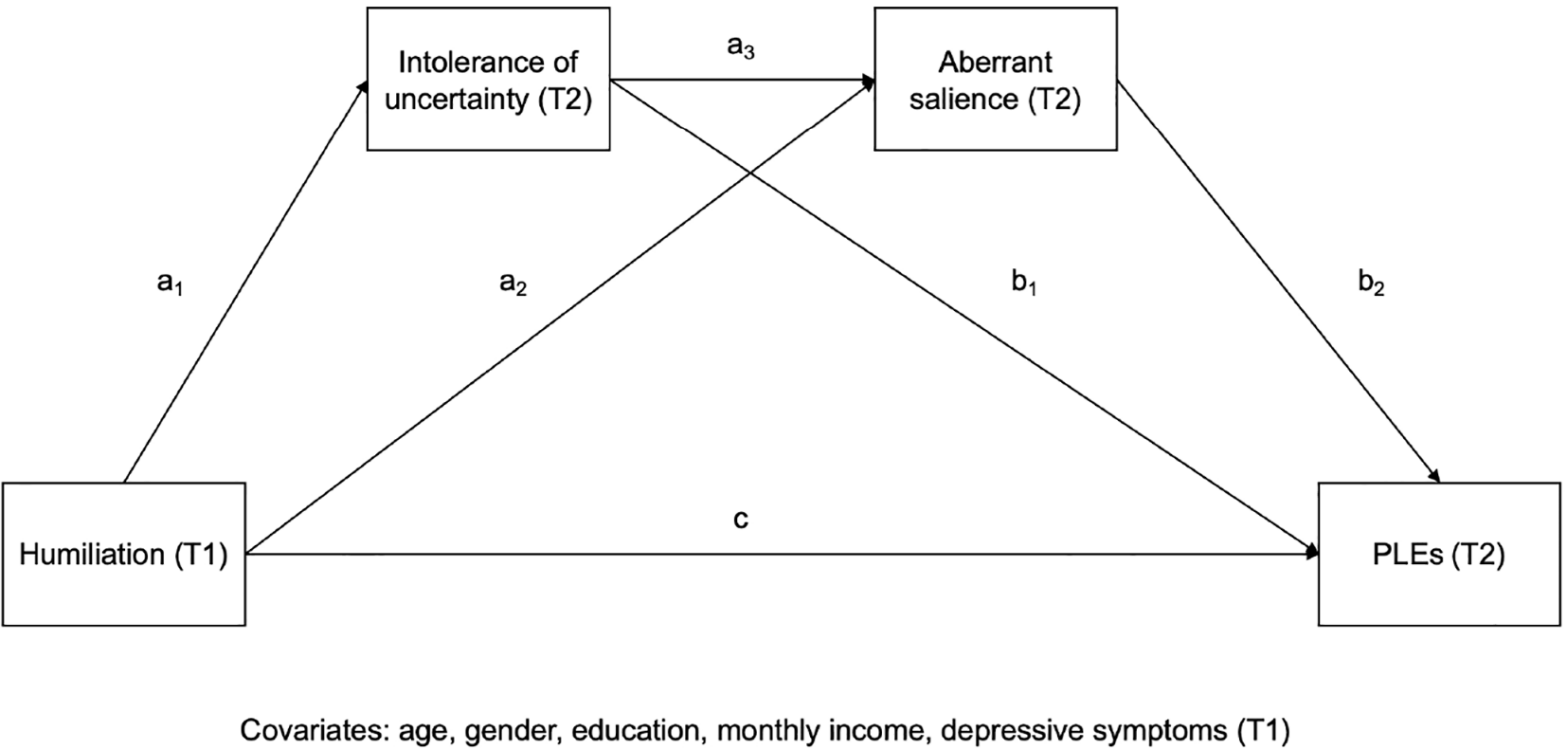
The serial mediation model tested in the present study. T1 refers to the first-wave assessment, and T2 represents the second-wave assessment. Paths a_1_–a_3_ and b_1_–b_2_ represent the indirect effects through the mediators, and *c* denotes the total (pre-mediation) effect of humiliation on PLEs.

The comparisons across continuous variables between participants completing both assessments and those lost to follow-up (here and after referred to as completers and non-completers) were performed using the Mann-Whitney U test (non-normal distribution) or t-tests (normal distribution). Normality of data distribution was assessed using the Kolmogorov-Smirnov test. Differences with respect to categorical variables were tested using the chi^2^ test. Bivariate correlations between humiliation, IU, AS, and PLEs were analyzed using the Spearman’s rank correlation coefficients due to non-normal distribution. In bivariate tests, results were interpreted as statistically significant in case of p < 0.05. After a series of bivariate tests, the PROCESS macro was used to assess serial mediation (model 6, see: **Figure 1**). The level of baseline humiliation (T1) was included as an independent variable (X) while the level of PLEs at the second wave (T2) was included as an outcome (Y). The levels of IU and AS (T2) were included as mediators (M). The models were analyzed before and after adjustment of covariates that included age, gender, the level of education, monthly income, and depressive symptoms (T1). Results were presented as standardized coefficients (β) with corresponding 95%CI values. Results were considered statistically significant if the 95%CI did not include zero. It was further explored whether mediation was full or partial (46). In mediation analysis, full mediation occurs when the association between an independent variable (X) and an outcome (Y) is entirely accounted for by one or more mediating variables (M), such that the direct effect of X on Y (controlling for M) is statistically non-significant, while the indirect effect via M is statistically significant. Partial mediation occurs when the indirect effect via M is statistically significant but the direct effect remains statistically significant as well, indicating that X influences Y both through the mediator(s) and through other pathways not captured by the model. All analyses were carried out in the SPSS software, version 28.

## 3 Results

Altogether, 4756 participants were invited to participate in the present study. In this sample, 1098 individuals (23.1%) reported a positive lifetime history of psychiatric treatment and 1417 individuals declined to participate or were non-responsive (29.8%). Therefore, 2241 individuals (30.3 ± 6.3 years, 53.4% females) completed the baseline assessment. At baseline, the participants were most likely to report a higher education level (49.4%), full-time work status (66.7%), and a monthly income equivalent to 750 – 1,500 USD (53.1%). From the initial sample (n = 2241), 1308 participants (58.4%) completed the second-wave assessment. Non-completers were younger, were more likely to be men (or reported gender identities other than men and women), had lower education levels, and reported unemployment status (see: **Supplementary Table 1**).

All constructs planned to be tested in a serial mediation model showed statistically significant and positive correlations (**Table 1**). Results of serial mediation analysis are reported in **Table 2**. The same effects were found statistically significant in unadjusted and adjusted analyses. After adjustment for covariates, the direct effect of humiliation on PLEs was not statistically significant (adjusted model: β = 0.045, 95%CI = –0.001 – 0.090). However, two indirect paths linking humiliation and PLEs appeared to be statistically significant. The first one led through AS (without a mediating effect of IU, (β = 0.062, 95%CI = 0.023 – 0.103). In turn, the second one led through IU and AS(β = 0.029, 95%CI = 0.018 – 0.042). Importantly, the indirect path with IU as the only mediator was not statistically significant(β = 0.003, 95%CI = –0.007 – 0.012). Also, direct effects of IU on PLEs were not statistically significant (β = 0.012, 95%CI = –0.033 – 0.056). However, statistically significant direct effects of IU on AS (β= 0.211, 95%CI = 0.153 – 0.269) and AS on PLEs(β = 0.643, 95%CI = 0.599 – 0.687) were observed. Altogether, the total effect, i.e., the sum of direct effect of humiliation on PLEs (β = 0.045) and total indirect effect (β= 0.094) was 0.139 in adjusted model. This means that 67.6% of the total effect of humiliation on PLEs was explained by indirect paths involving IU and AS. Descriptive statistics for the variables used in the mediation analyses are shown in supplement (see: **Supplementary Table 2**).

**Table 1:** Bivariate correlations between constructs assessed in the present study.

	Humiliation	IU	AS
Humiliation	–		
IU	r = 0.343, p < 0.001	–	
AS	r = 0.307, p < 0.001	r = 0.339, p < 0.001	–
PLEs	r = 0.319, p < 0.001	r = 0.306, p < 0.001	r = 0.724, p < 0.001

AS, aberrant salience; IU, intolerance of uncertainty; PLEs, psychotic-like experiences.

**Table 2:** Results of a serial mediation analysis with adjusted covariates and unadjusted mediations.

Effect	Path	Unadjusted analysis			Adjusted analysis^*^		
		β	95%CI		β	95%CI	
			LLCI	ULCI		LLCI	ULCI
Humiliation → IU	a_1_	0.342	0.291	0.393	0.214	0.156	0.273
Humiliation → AS	a_2_	0.216	0.163	0.269	0.097	0.038	0.157
IU → AS	a_3_	0.264	0.211	0.318	0.211	0.153	0.269
IU → PLEs	b_1_	0.043	0.002	0.084	0.012	–0.033	0.056
AS → PLEs	b_2_	0.680	0.640	0.720	0.643	0.599	0.687
Humiliation → PLEs	c	0.004	–0.004	0.013	0.045	–0.001	0.090
Humiliation → IU → PLEs	a_1_b_1_	0.015	–0.002	0.028	0.003	–0.007	0.012
Humiliation → AS → PLEs	a_2_b_2_	0.147	0.107	0.189	0.062	0.023	0.103
Humiliation → IU → AS → PLEs	a_1_a_3_b_2_	0.062	0.047	0.079	0.029	0.018	0.042
Total indirect effect	ab	0.223	0.181	0.266	0.094	0.053	0.136

Statistically significant effects refer to those where 95%CI does not include zero. Age, gender, education, monthly income, and baseline depressive symptoms were included as covariates. AS, aberrant salience; IU, intolerance of uncertainty; PLEs, psychotic-like experiences.

## 4 Discussion

The main findings of the present study suggest that IU and AS might mediate the association between humiliation and PLEs. Mediation was observed for two paths, i.e., through AS as well as through IU and AS. Given that the direct effect of humiliation on PLEs was not statistically significant, it might be concluded that both paths appeared to fully mediate the association of humiliation with PLEs. The serial mediation model included IU and AS simultaneously to control for shared variance between these constructs. This modeling approach allowed to test the independent mediation effects of each construct on the relationship between humiliation and PLEs. Hence, the statistically significant indirect effects identified reflect the unique contribution of each mediator, supporting the specificity of IU and AS as distinct psychological pathways. These results are supported by findings from previous studies indicating the association of low social status with heightened awareness of threat and development of psychotic symptoms (6, 47, 48). Low social status is often linked to, and discussed in the context of, the experience of cumulative humiliation (6, 10, 42, 49). The relationship between low social status (and thus humiliation) and the development of psychotic symptoms has been described and studied within the CSA concept (48). Our previous study found that humiliation might be the CSA agent most closely related to the development of PLEs, through the mediating effect of AS (11). Our present study extends the CSA hypothesis by demonstrating that the relationship between humiliation and PLEs may be specifically mediated not only by AS, but also to a lesser extent by IU. Nevertheless, caution should be exercised when interpreting this conclusion, as the limited duration of the observation period in this study prevents definitively establishing causality within the serial mediation model.

In accordance with findings from the present study, we hypothesize that the experience of humiliation may heighten the individual’s threat perception and responsiveness, thereby lowering the threshold for perceiving ambiguous stimuli as threatening or distressing, creating cognitive processing differences. Thus, IU might be considered a cognitive pattern (or ‘emotional state’ derived from cognitive interpretive tendency) that partially accounts for the relationship between cumulative humiliation and the development of salience alterations. In line with this reasoning, the study conducted by Demirtas and Yildiz (2019) found that IU is negatively associated with ‘cognitive flexibility’, defined as the capacity to shift between different cognitive sets in order to adapt to dynamic, environmental conditions (50, 51). In other studies, low cognitive flexibility has been linked not only with IU, but also with perceived stress, affective disturbances, reduced ability to control disturbing thoughts, and hopelessness (50–52). Moreover, a recent study revealed that cumulative humiliation and the state of anxiety jointly predicted cognitive-perceptual disturbances and PLEs among healthy individuals (53). These results suggest that experiencing CSA may foster not only the development of psychotic symptoms and affective disturbances (9, 11, 48), but also cognitive processing patterns, for example IU or AS (11, 50, 53–55).

Our study demonstrated that while AS alone may be sufficient to mediate the association between humiliation and PLEs, IU alone appears insufficient to do so. The neurobiological and clinical link between AS and PLEs is well established (35, 56), and the observed mediating role of AS aligns with our predictions. Although research suggesting IU as a potential risk factor for psychosis has expanded in recent years, this topic requires further empirical validation (29). Existing literature indicates that IU is more strongly associated with specific psychotic symptoms, particularly those linked to negative affectivity, than with perceptual abnormalities (19). This may help explain our findings, in which the direct effects of IU on AS were more pronounced than its direct effects on PLEs. We hypothesize that IU may represent a psychological processing pattern shaped by prolonged experiences of cumulative social adversity, which affects and co-occurs with heightened negative affectivity, depression and anxiety (19, 29). Repeated experiences of humiliation and sustained low social status may heighten uncertainty about the future and intensify the anticipation of threat, given that low status is frequently associated with poorer health, higher morbidity rates, and increased risk of mental disorders (57–59). These processes could prime the perceptual system to detect and assign significance to ambiguous stimuli, thereby promoting AS and, in turn, contributing to PLEs (5, 14, 16, 60). This pattern suggests a hierarchical relationship, i.e., IU may primarily integrate symptoms related to negative affectivity and paranoid beliefs (19), AS may integrate both affective and perceptual elements that lead to PLEs, and PLEs themselves predominantly reflect perceptual abnormalities. Distinguishing these pathways might be essential for clarifying the specific psychological mechanisms through which different constructs contribute to various prodromal symptoms.

Taking into account previous studies on decision-making deficits across the psychosis spectrum, it is needed to note similar theoretical considerations around another cognitive processing pattern known as jumping to conclusions (JTC) that can be defined as interpretations or judgments that are made early and in response to insufficient evidence (60). Findings from some studies suggest that individuals prone to psychosis also show a specific hasty decision-making style, requiring less information to come to a conclusion compared to healthy controls (55). Importantly, IU has been posited as the phenomenon motivating early termination of data gathering thereby promoting the occurrence of JTC. Our findings warrant the discussion about potential mechanisms explaining the association of humiliation with various cognitive patterns and PLEs.

Finally, existing empirical evidence indicates a relationship between anxiety, depression, and PLEs, demonstrated through mediation analyses (8, 9, 61, 62). This process is commonly referred to as the affective pathway to psychosis (63). Our previous work supported this framework, showing that both depressive symptoms and AS mediated the relationship between humiliation and PLEs (11). In the current study, we considered IU in the context of its established links to anxiety and negative affectivity (12, 64). While our data do not allow firm conclusions about its causal role, the affective implications of IU may offer an additional perspective for understanding how repeated experiences of humiliation could contribute to salience alteration and, in turn, the development of PLEs. The findings of Toh and colleagues (2024) are consistent with this broader view, highlighting the combination of humiliation and anxiety as a potential contributor to PLEs. We suggest that IU could be explored further in future research on affective pathways to PLEs.

Our results suggest that humiliation and AS, and to a lesser extent IU, might be involved in the development of PLEs. This process could potentially lead to the clinical onset of psychosis; however, this line of reasoning requires further investigation. This assumption is based on both theoretical and empirical evidence, indicating a pathogenetic pathway leading to psychosis via PLEs, AS, and potentially humiliation and IU (3, 11, 28, 65). We consider these factors as causes rather than consequences of early psychosis development. An alternative hypothesis, that early psychotic symptoms increase sensitivity to humiliation, IU and AS, does not account for the presence of IU and AS in individuals without psychosis, including those at familial or environmental risk (27, 29). Moreover, humiliation being externally observable, often occurs before the onset of psychiatric symptoms, particularly during formative developmental periods (e.g. adolescence), making a reverse temporal order less comprehensible (6, 42).

Limitations of the current study should be considered when interpreting the results. The sample was not assessed through clinical interviews to record the presence of underlying psychiatric disorders. Nevertheless, studies indicate that even self-reported PLEs, revealed as false positives after a comprehensive clinical assessment, may still predict the onset of psychosis (65). Furthermore, clinical relevance of observed associations might be limited, as the study did not include clinical populations. Moreover, statistically significant effects observed in the present study were generally small (except for the large effect of AS on PLEs). However, the study was based on a non-clinical sample in order to capture the emergence of PLEs and avoid the confounding of psychiatric treatment. Another limitation is a short period of observation with only two waves of assessment. Therefore, temporal ordering based on a serial mediation model cannot be clearly concluded. Moreover, representativeness of the sample is difficult to assess, as specific reasons underlying non-participation were not recorded. In addition, it is important to note that non-completers had significantly higher levels of AS and PLEs. Additionally, they differed significantly in their sociodemographic profiles compared to those who completed the study. Future research should be based on longitudinal studies with at least three waves or systematic data collection methods that measure the level of humiliation, IU, AS, and PLEs.

Our analysis may provide a more nuanced understanding of how social and cognitive processes interact to contribute to the emergence of PLEs in otherwise healthy individuals. The findings suggest that cognitive processing patterns, such as AS and IU, may serve as key psychological mechanisms through which experiences of humiliation promote the development of PLEs. Specifically, intense feelings of humiliation may prime the perceptual system to detect and attribute heightened significance to ambiguous stimuli, thereby increasing uncertainty and, ultimately, contributing to the occurrence of PLEs. Future studies might therefore consider IU as a novel indicator of the CSA hypothesis, given that IU may enhance the development of AS and, in turn, facilitate the onset of PLEs. Further investigation of the CSA hypothesis could also determine which cognitive processing patterns most strongly reinforce the development of PLEs.

From a clinical perspective, the findings highlight the importance of addressing cognitive processing differences during non-pharmacological interventions, particularly therapeutic approaches that target heightened levels of AS. While reducing AS may remain a primary objective of psychological treatment, our results indicate that targeting IU may also be beneficial, as it could influence AS levels. This holds significance, given that cognitive behavioral therapies commonly employed for anxiety and depression have demonstrated efficacy in mitigating intolerance of uncertainty (64). Moreover, we emphasize the potential value of early psychological interventions aimed at mitigating exposure to prolonged humiliation in order to prevent the emergence of PLEs in vulnerable individuals. Taken together, these findings underscore the potential of combining interventions to enhance treatment efficacy.

## Data Availability

The original contributions presented in the study are included in the article/**Supplementary Material**. Further inquiries can be directed to the corresponding author.
