# Supplementary material for: The cumulative social adversity hypothesis of psychosis: Intolerance of uncertainty and aberrant salience mediate the association between humiliation and psychotic-like experiences
Source: Front Psychiatry. 2025 Nov 7;16:1647155. doi: 10.3389/fpsyt.2025.1647155 (PMC12634645; doi:10.3389/fpsyt.2025.1647155)
Supplement: Supplementary file 1 [file Table1.docx]

**Supplement Table 1.** Descriptive characteristics of the sample.

|  | Completers,  *n* = 1308 | Non-completers,  *n* = 933 | *p* |
| --- | --- | --- | --- |
| Age, years | 31.1 ±5.9 | 29.1 ±6.6 | < 0.001 |
| Gender  Men  Women  Other | 627 (47.9)  677 (51.8)  4 (0.3) | 413 (44.3)  520 (55.7)  0 (0) | 0.049 |
| Education  Primary  Vocational  Secondary  Higher | 23 (1.8)  75 (5.7)  489 (37.4)  721 (55.1) | 41 (4.4)  86 (9.2)  419 (44.9)  387 (41.5) | < 0.001 |
| Unemployment, yes | 153 (11.7) | 146 (15.6) | 0.008 |
| Income^*^  < 750 USD  750 – 1,500 USD  1,500 – 2,500 USD  2,500 – 3,750 USD  > 3,750 USD | 317 (28.0)  605 (53.5)  174 (15.4)  27 (2.4)  8 (0.7) | 234 (31.5)  391 (52.6)  93 (12.5)  17 (2.3)  9 (1.2) | 0.218 |
| Depressive symptoms | 7.9 ±5.7 | 8.4 ±5.5 | 0.059 |
| Humiliation | 27.0 ±12.2 | 27.0 ±12.1 | 1.000 |
| IU | 37.5 ± 8.9 | – |  |
| AS | 9.0 ± 7.9 | – |  |
| PLEs | 3.0 ± 3.5 | – |  |

Data are shown as mean ± SD or n (%)

^*^Missing data in case of 177 completers and 189 non-completers

*Note:* AS, aberrant salience; IU, intolerance of uncertainty; PLEs, psychotic-like experiences

**Suplement Table 2.** Descriptive statistics for variables included in the mediation analysis.

|  | **Mean** | **SD** | **Skewness** | **Kurtosis** | **N** |
| --- | --- | --- | --- | --- | --- |
| X | 27 | 12,18 | 0,65 | -0,39 | 2241 |
| M_1_ | 9,02 | 7,88 | 0,82 | -0,21 | 1308 |
| M_2_ | 37,47 | 8,93 | -0,38 | 0,45 | 1308 |
| Y | 2,97 | 3,53 | 1,47 | 1,6 | 1308 |

*Note:* X - cumulative humiliation; M_1_ - aberrant salience (AS); M_2_ - intolerance of uncertainty (IU); Y - psychotic-like experiences (PLEs)
